# Hospital and Institutional News

**Published:** 1912-07-06

**Authors:** 


### HOSPITAL AND INSTITUTIONAL NEWS.

### VOLUNTARY HOSPITALS AND THE INSURANCE ACT.

We publish to-day an interesting discussion, which took place in Glasgow on June 28, on several questions relating to the Insurance Act which very closely affect all voluntary hospitals. We report this discussion fully, because the points are of the utmost importance, and they reveal the hopeless unpreparedness of the Commissioners and other authorities charged with the duty of applying the Act which is to come into force on the 15th instant. First, there is the question of tuberculosis and to what extent the voluntary hospitals will be able and willing to avail themselves of the Act by taking *pro rata* payments, with a sum for medical attendance, for all cases of tuberculosis treated in their wards. Then there is the question as to what course the voluntary hospital managers are to take in regard to the admission of insured persons, where the members of their medical staff have resolved not to treat such patients unless or until the medical profession and authorities have agreed to work together; the effect which the Act will have upon the medical treatment of members of the nursing staff by the hospital authorities, and the provision, if any, which will have to be made for the domestic staff of each voluntary hospital. The position of Poor-Law infirmaries, fever hospitals, and other rate-aided institutions *vis-à-vis* with the Act is another important aspect of national insurance. Most of these questions were raised at the Glasgow Conference, and they will have to be faced and clearly understood by the managers of every voluntary hospital if many difficult points which arise are to be wisely solved. In referring our readers to the Glasgow report we are struck with the evidence it affords once again of the apparent paralysis which is gradually overtaking the English people, who seem to have lost the energy required to protect their own interests, not only in regard to national questions, but in matters which affect them so closely as national insurance. Either English men and women, whether connected with our hospitals or as mere residents in that portion of Great Britain called England, must wake up speedily and assert their rights or their inertness and incapacity for self-protection may speedily bring these islands to the condition of impotence which precedes the disappearance of nations through over-civilisation and the possession of too much luxury. King George knew what he was about when at the Mansion House he uttered the momentous warning, "Wake up, England!" We are glad to note that Scotland at least is awake in this matter of the Insurance Act.

### A PHYSICIAN'S CLAIM TO A PEERAGE.

Some twelve months ago three claimants came forward to the Baronies of Burgh, Strabolgi, and Cobham. One of these is Dr. Reginald Gervase Alexander, of Blackwall Lodge, Halifax, consulting physician to the Bradford Royal Infirmary and to the Halifax Royal Infirmary, at both of which he had formerly held the post of senior physician. Dr. Alexander graduated at Cambridge in 1874, and took the M.D. of Edinburgh in 1881. His publications include "Phthisis: Its Prevention and Cure" and "The Art of Prolonging Life." Dr. Alexander has two sons and two daughters; one of the former is in the medical profession. His claim is now under consideration by the Committee of Privileges. This august body, which in the past has often given classic examples of the law's delay, is inclined, however, to pale in importance in the minds of all who have read Mr. J. Horace Round's masterly and delightful "Peerage and Pedigree." It is he, readers of his works will feel at once, who really should decide such issues, for his is the gay spirit which, as a petulant lordling was once heard to remark, "if it cannot deprive us of our heirlooms at any rate it deprives us of our ancestors." Dr. Alexander's claim, we trust, will prove too well-founded even for "the curious eye" of the Committee of Privileges, to which, we believe, Mr. Round is the expert adviser.

### AGGRIEVED ARCHITECTS.

A blunder which can be amusing to none, and to many architects is causing grave annoyance, has occurred in connection with the projected sanatoria of the Welsh anti-phthisis campaign. The circumstances are briefly these. On May 31 the executive advertised inviting architects to submit designs by June 24 for sanatoria to contain respectively 250 and 150 beds. On June 10 an urgent letter was addressed to competitors extending the date to July 8, and on June 27 a second letter admonished competitors in pleasant words that shortly further particulars, so far-reaching as to form a basis for a fresh competition, would be advertised. Thus many architects have been invited to spend a whole month of serious work on a difficult problem for which no satisfaction or compensation is forthcoming. We are surprised that the Royal Institute of British Architects did not take some stand in the matter to protect its members, for meanwhile an embarrassing situation may develop in the legal relation of the parties. One indignant architect has suggested that the campaign is proving more fatal to architects than to tubercle bacilli at the moment. And, petulance apart, a serious blunder has been committed. Who is the culprit? The hardship of it all is that it is very doubtful whether the architects who have wasted so much time and energy have any legal redress. In an old case a learned judge said: "It is of everyday occurrence for architects to send in plans for public buildings, taking the chance of being paid for their labour or not as they may be adopted or rejected." This rule holds good in the case of an ordinary competition; but there does not appear to be any case which defines the rights of competitors who, through a modification of the terms of the competition, have expended a large amount of time and trouble in preparing plans for which they do not even run the chance of being remunerated. It would be very interesting to see the result of a test action brought by an architect who felt himself aggrieved in these circumstances.

### THE ROYAL VISIT TO CARDIFF.

That portion of the Royal visit to Cardiff which is not described in detail in the two special articles which we publish this week concerning their Majesties' inspection of the King Edward VII. and of the Hamadryad Hospitals deserves separate notice, for it included a visit to the University College of South Wales and Monmouthshire. On arriving at the south gate of the College the King and Queen were received by the Principal, Dr. E. H. Griffiths, and by the Chief Marshal, Professor D. Hepburn, Dean of the Faculty of Medicine, who were presented by the President and Vice-President of the Students' Representative Council, Mr. Henry Lewis. The architect, Mr. W. D. Caröe, M.A., was also presented, and the King and Queen made their way through ranks of students to the Committee Room. The Viriam Jones Research Laboratory was then inspected, and after leaving the Council Chamber a procession was made to the Drapers' Library, in which Professor A. H. Trow, Dean of the Faculty of Science, Mr. Percy E. Watkins, Registrar, and Mr. W. T. Edwards, M.D., Vice-President, took part, and were then presented to their Majesties. After leaving the College the King and Queen returned to the Royal yacht, and spent the afternoon in visiting Llandaff Cathedral and the Royal Hamadryad Seamen's Hospital. Of their visit to the latter a full account appears elsewhere. The Chairman of the Hamadryad Hospital, Mr. John Moore, the Vice-Chairman, Mr. William Jones, and Dr. Whelan had the honour of being presented to the King and Queen by the Lord Mayor of Cardiff at the hospital.

### TWO GREAT HOSPITAL SCHEMES FINANCED.

Two munificent gifts have to be chronicled this week, and both of them are from anonymous donors. First, in appreciation of the visit of their Majesties the King and Queen to the King Edward VII. Hospital, Cardiff, a benefactor, as we note elsewhere, has sent ten thousand guineas for the building of the new pathological wing of the institution, erected for the Welsh Medical School as the joint establishment of the Hospital and University College of South Wales and Monmouthshire. It is estimated that £50,000 will be required to complete the medical school buildings. Secondly, the Treasurer of St. Thomas' Hospital, London, was able to announce on Thursday of last week that a South Londoner has promised him the sum of twenty thousand pounds towards the building of the new out-patient department which the hospital authorities regard as imperatively necessary. With such a splendid nucleus there should be no insuperable difficulty in making up the full sum required for this purpose, estimated at forty to fifty thousand pounds.

### THE EXTENSION OF WINDSOR HOSPITAL.

Two generous offers have just been made for the provision of additional accommodation at King Edward VII. Hospital, Windsor. We understand this is much needed, and in consequence both of these have been accepted gladly by the Committee. The first is from Mr. A. W. Mayo Robson, C.V.O., for a new and larger children's ward to replace the present one in temporary use. The change will also permit of additional space being rendered available for the *x*-ray work and for electrical treatment, an important consideration in the eyes of the medical staff. The second is from Mrs. James Elliman, who offers to give a special ward for the surgical treatment of women patients. Apart from the cost of building, which is estimated at about £2,600, Mrs. Elliman has promised £2,000 towards its endowment and £400 towards the improvement and extension of the electrical department. Each ward unit will include a sister's room, heating apparatus, a kitchen, linen store, and bathroom. The plans for the new wards are in an advanced stage, and their erection will probably be proceeded with during the present summer.

### WANTON NOISES IN HOSPITALS.

A very simple and sure test of the relative efficiency of a hospital is freedom from avoidable noise. We noted recently that the patients who were really ill in the wards situated on the ground floor of a great hospital were wantonly disturbed by the exasperating and unnecessary noise caused by permitting the porters to wheel milk cans on trolleys without rubber tyres along the basement corridors near the wards. Some of the patients who were very ill suffered acutely two or three times a day from this cause, which revealed at once an absence of supervision and an indifference to efficiency which surprised us. In a nursing home for paying patients there was more noise than it would be possible to find probably in any other establishment, even in London. In serving the meals the amount of noise created by throwing the knives and forks about and handling the dishes and other necessaries of the service was almost incredible. These things should not be. Every matron of a hospital and every sister of a ward who has any claim to be efficient should make it her business to prevent noise to the fullest possible extent. In a large ward there must sometimes be unavoidable noises. Where, however, the matron of an institution possesses the true spirit of service her sisters and herself co-operate together to reduce noise to a minimum, and to minimise everything calculated to disturb the patients who are really ill, no matter how large the ward may be. We should be glad to have the experience of workers and patients in regard to noise in hospitals. Our experience leads us to think that a hospital often attains an undesirable local reputation from the absence of due care for the feelings of the patients in regard to noises of all kinds. Members of hospital committees might usefully make it their business to visit the wards of their hospital at various hours, to note especially whether this point has due attention, and to treat the absence or presence of noise of the kind referred to as evidence of the efficiency or otherwise of their staff. In this way an immensity of good might be done in certain hospitals, especially where some of the staff may be mere martinets, who put routine before everything.

### A FRIENDLY HINT TO COL. LOCKWOOD, M.P.

On Thursday last week Colonel Lockwood, M.P., opened the Epping Forest Hospital. Its foundation-stone was laid by Sir T. Vezey Strong when Lord Mayor of London in September last. The building has cost £3,300; the equipment, not counting several valuable gifts, £400; and the site, £375. A total of £4,250 has been spent, towards which donations have come in for about £4,000. The Rev. Dr. Woodward, chairman of committee, presided, and Mrs. Buxton, Mr. W. Hudson, hon. secretary, and Mr. Bennett Wood, assistant secretary, were present. The Rev. Arthur Montford, rector of Loughton, was followed by the hon. secretary, who remarked that Mr. E. North Buxton, J.P., C.A., had sent £50. Colonel Lockwood said that was the first time he had ever taken part in the opening of a hospital, and he then opened the door with a golden key handed to him by Master Crossman. For the benefit of Colonel Lockwood we may add that practically everything which could be said intelligently on such occasions as the laying of hospital foundation-stones, the opening of completed hospitals, and the inauguration of hospital convalescent homes was said, and on the whole very well said, by the late Mr. Passmore Edwards, who must have played the part which Colonel Lockwood undertook for the first time last week, more often than most men, even than that modern man of functions, the paid M.P. Fortunately, Mr. Passmore Edwards' speeches have been preserved in book form, and may be commended to those gentlemen, who, like Colonel Lockwood, are somewhat diffident at their first attempt at opening a hospital.

### THE SUNDAY FUND WEEK BY WEEK.

Up to Saturday last about £38,000 had been received from the collections at London churches and chapels on Hospital Sunday. Among them we note especially the first six: Christ Church, Lancaster Gate, heads this list with £949; Holy Trinity, Sloane Street, comes second with £408. The general grounds of the drop between its collection and that of Mr. Gurdon's congregation will best be appreciated by those who had the privilege of reading what the Vicar of Holy Trinity, Sloane Street, Mr. H. R. Gamble, wrote in The Hospital Sunday Special Number, which was placed in the pews of every church on Hospital Sunday. Third comes St. Peter's, Eaton Square, with St. John's, Wilton Road, £392; fourth, Kensington Parish Church and sister churches, £376; fifth, St. Mark's, North Audley Street, £257; sixth, St. Peter's, Cranley Gardens, £212. On page 370 will be found the remaining names and totals.

### ACCOMMODATION CONTRACTS FOR LONDON'S MENTAL PATIENTS.

Notice has been given by the committee of visitors for Brookwood Mental Hospital to determine, on September 30 next, the contract under which 25 London women patients are maintained there. The following contracts have recently been entered into:—Brighton Borough Mental Hospital, Hayward's Heath, for 20 males until 1917, at 14s. a week each; East Sussex County Mental Hospital, Hellingly, 10 males until 1917, at 15s. a week; West Sussex County Mental Hospital, 20 males until 1917, at 15s. 2d. a week; and Oxford County Mental Hospital, Littlemore, 20 males until 1917, at 14s. a week. The Oxford contract has not yet received the approval of the Secretary of State. The Mental Hospitals Committee of the London County Council are endeavouring to secure contracts for still further accommodation.

### ACCOMMODATION FOR POOR MENTAL PATIENTS.

The annual report of the General Board of Commissioners in Lunacy for Scotland which has just been issued shows that on January 1 of the present year there were 19,034 (an increase on the previous year of 398) mentally afflicted persons, of whom 2,608 were maintained from private sources, 16,371 by parochial rates, and 55 at the expense of the State. In Royal mental hospitals there was an increase of 32 private patients and of 53 "pauper" patients; in district mental hospitals there was an increase of 10 private patients and of 216 "pauper" patients; in private mental hospitals there was a decrease of 5 in the number of private patients; while in the mental wards of the poorhouses there was an increase of 27 "pauper" patients. In a chapter on want of accommodation for the poorer class of private patients the report expresses satisfaction that proposals suggested in a previous report have been embodied, with slight modifications, in one of the clauses of the new Amending Lunacy Bill (Scotland) now before Parliament. The main proposals were that District Lunacy Boards should be authorised to receive private patients, and also to provide accommodation for such patients, if they should see fit, by erecting separate buildings or by setting apart for the purpose sections of existing buildings; and that the rate of board chargeable for maintenance should not be higher than the maintenance rate charged for "pauper" patients, with the addition of a sum in name of rent for the accommodation afforded. The object of these proposals is to render the position of private patients in district mental hospitals more secure, and to encourage an increasing and deserving class of the mentally afflicted to become private patients at low rates of board.

### A LOSS TO MARLBOROUGH.

Dr. Oliver Calley Maurice, of Marlborough, the locally well-known son of the late Dr. J. B. Maurice, the Marlborough memorial to whom we referred to recently, has died suddenly of pneumonia at the early age of forty-two. He was a student at St. Mary's Hospital, London, and held the conjoint qualification from the year 1893. After acting as assistant medical officer to the Marylebone Infirmary, Dr. Maurice took part in his father's practice at Marlborough. He held many appointments in the vicinity, including those of surgeon to the Savernake Cottage Hospital, district medical officer, and public vaccinator. Dr. Maurice was also a surgeon-major in the Royal Wilts Yeomanry and took an active part in the work of the National Defence League. To all medical men who realise what the building up of a cottage hospital really entails, and who care to see preserved the best traditions of English country practice, the double loss which the profession generally, and especially its branch at Marlborough, has sustained will be appreciated at its true value.

### MORE STAFF VACANCIES IN LONDON.

Not long ago a comment was evoked in these pages (The Hospital, June 1, p. 217) by the unusual number of vacancies on the honorary visiting staffs of the London hospitals. After such a glut of appointments, it might reasonably be supposed that chances would be scarce for some time. But, in point of fact, there seems to be a second crop of them. Thus at the present date an assistant surgeon is required at the Dreadnought Hospital, Greenwich; a physician to out-patients at the City of London Hospital for Diseases of the Chest, Victoria Park; an assistant physician at the London Skin Hospital, Fitzroy Square; a surgeon and one, or possibly two, assistant surgeons at the West London Hospital, Hammersmith; and two assistant physicians at the National Hospital for the Paralysed and Epileptic, Queen's Square. The death of Dr. Murrell will of course entail a vacancy or series of vacancies at the Westminster Hospital.

### ACTON ISOLATION HOSPITAL EXTENSION.

To what an extent the pressure at a fever hospital differs from that of an ordinary hospital is shown by the fact that over 57 per cent. of the cases treated in Acton Isolation Hospital last year were admitted during the last four months, and 46 per cent. during the last three months. With the opening of a new pavilion, which has cost £4,159, or 4.66d. per cubic foot, the hospital can now accommodate 76 patients. The new building is divided into two wards of eighteen and sixteen beds respectively, and two separation wards, each of which has accommodation for one patient. Leading out of each ward, in the centre of the building, is a bathroom, and at the extreme end are the sanitary annexes. The building is heated with hot water on the low-pressure system, the cost of the heating apparatus being £668, and the steam for heating the water in a calorifier being conveyed from the refuse destructor. In each ward there are Shorlands ward ventilating stoves, but these are only used when it is inconvenient to heat with steam.

### THE "NOBILITY" OF MEDICINE.

The majority of medical men, fortunately for their self-respect, understand too well what are the real virtues and glories of their profession to care very much to have it called "noble" by the layman. For the layman is usually a man who calls a doctor noble in order to get him out of bed in the middle of the night, only to abuse him as avaricious and inhuman, if the doctor pauses on his doorstep to inquire what chance there is of his gaining his fee. If the doctor does not inquire for it, outside the little oasis of West-end practice, he is despised for the inevitable insolvency which awaits an excessive indulgence of his humane instincts. If he does ask for it in advance, no name is too bad for him. That is why most members of the medical profession will skip a lay article this week on "The Nobility of Doctors," thanking Mr. James Douglas meantime for his well-meant eulogia. The moral really is that every profession is or can be noble in its own way, and that men who work as hard as doctors, and who have their chance of imbibing the scientific spirit, see more clearly than their fellows, particularly than those engaged in the weekly praise of other men's books, the idleness of attaching fine names to the day's work, and are as much above the vulgar need or desire for gratitude as the doctor was in the romantic episode related in Mr. James Douglas' article.

### HOSPITAL TOURS FOR HOSPITAL OFFICERS.

The Medical Study Tour of the ninth session of the Association Internationale de Perfectionnement Scientifique (Scientific Improvement International Association), patronised by the French Government, has been organised by the Central Board, and the prospectus, after stating the itinerary, which starts from Besançon and includes such places as Salzburg, Bucharest, Constantinople, Belgrade, and Venice, after four weeks of travel ending on August 30 at Aix-les-Bains, states: "The tour will take place on the best conditions in every respect and without the least trouble, as the Sections of the 'A.P.M.' at the Balkans and Constantinople have prepared the organisation of same with the greatest care. An illustrated and fully detailed programme will be published." This, at least, shows large ideas, and presupposes "the greatest care" in its organisation. Why, it may be asked, cannot a British organisation invoke the aid of hospital workers on the Continent to produce a similar programme and to achieve, or at any rate, to offer, a similar tour? Hospital workers of all grades travel more every year, even if they cannot really be said to travel enough, and an organisation on these lines would be worth attempting. Mr. Conrad W. Thies for years has set a valuable example by his travels. He would be the right man to appeal to, also, on points of organisation, and practical difficulties.

### THE ADMISSION OF PATIENTS IN EXTREMIS.

The Wandsworth Board of Guardians have been somewhat surprised at a statement in the annual report of the medical superintendent of St. James's Infirmary that during the last year forty-nine cases were admitted in extremis, the patients dying within twenty-four hours of admission. The matter was referred to the visiting committee for consideration, and, after they had gone through the observations of the district medical officers and the relieving officers, they reported to the Board that they were satisfied. Dr. E. A. Miller did not concur with the committee. He was of opinion that in some cases the patients need not have been removed from their homes, whilst in others they ought to have been admitted to the infirmary long before. He proposed that the medical and relieving officers should make a fortnightly return of patients dying within twenty-four hours of their admission to the infirmary, with their observations on the cases. The Board, by twenty-one votes to five, adopted the resolution.

### WHAT CONSTITUTES A HOSPITAL TRAINING SCHOOL FOR NURSES?

The answer to this question is not so simple as it sounds, as the following incident will show. The chairman of a certain hospital which possesses over 100 beds in the aggregate, but which is not housed under contiguous roofs, is constantly met with a difficulty when advertising for probationers, who reply with alarming unanimity that it is not a recognised training school. By what right, the authorities are beginning to ask, is this apparently arbitrary distinction drawn in its disfavour? Who laid down the rule that 100 beds, and no fewer, were necessary for a hospital to be regarded as a training school, and why was this particular figure chosen? The answer to the first of these two questions is that the examining bodies laid down many years ago the rule that in London a hospital claiming recognition as a training school for medical students must have not fewer than 150 beds, and in the provinces not fewer than 100. The rule that applies to medical students applies with equal force to nurses. Besides, a moment's reflection will show that the principle involved, like many a wise proverb, ultimately stands on its transparent common sense. The number 100 is not sacrosanct in itself, but was obviously chosen as representing in a simple but most practical manner a certain status, organisation, and body of work. These are all essentials to the efficient training of medical students and nurses, and are guaranteed, humanly speaking, by an institution the beds of which run into three figures. But in order to guarantee that the spirit represented by a dash and two noughts shall not be sacrificed to the letter of evasion, a proviso is added that these beds must be housed under one or a contiguous congeries of roofs.

### A ROMANTIC BEQUEST.

Alderman W. C. Lupton, the sole surviving trustee of the East Morley and Bradford Savings Bank, which some years ago sold its investment business for £10,500—this sum not being available for distribution among the shareholders—was warned with his co-trustees that they could divide it among local charities only at their peril, with the result that the money was therefore left in the bank. Last week, however, Mr. Lupton was able to announce that, though he had been again warned, "the warning was accompanied by a smile." He had, therefore, decided to divide the original sum and accumulated interest among charities in Bradford, Harrogate, Ilkley, and district. He gave £3,500 to Bradford Infirmary, £1,000 each to the Bradford Eye and Ear Hospital and the Bradford Children's Hospital, besides various smaller sums to other charities. These had been satisfied, Mr. Lupton explained, in respect of £11,500 by the transfer of Consols, and £150 had been paid in cash, making the total distribution £11,650. He added that with a considerable portion of the money, about four years ago, Consols were purchased at 111, which to-day had fallen to 76½, and they could reckon up what had been lost.

### THE RAYMOND HORTON-SMITH PRIZE.

In awarding the Raymond Horton-Smith Prize for 1912 to Dr. V. J. Woolley, the Cambridge University M.D. Degree Committee place on record certain views with regard to the researches submitted which must be gratifying both to the Regius Professor of Medicine, Sir T. Clifford Allbutt, and to Mr. Horton-Smith, K.C. The Committee state that they appreciate the high standard attained by most of the theses submitted for the Doctorate of Medicine at this University. Many of them consist of investigations into obscure diseases or of original laboratory research, and should, in the opinion of the Committee, be published. Besides the prize-winner, Dr. A. E. Barclay is nominated as *proxime accessit*; and Dr. G. G. Butler, Dr. A. J. Clark, and Dr. F. P. Franklen-Evans receive special distinction. It is noteworthy that all but one of the theses thus commended deal with pathological or laboratory, rather than strictly with clinical, research. This, possibly, may be due to certain express stipulations in the original foundation. The University could very well do with a similar benefaction for the encouragement of more especially clinical studies among aspirants for the doctorate. The award of higher degrees by the thesis system instead of by examination has been scoffed at; and it is, of course, susceptible of grave abuses if the authorities wink at them. But the success of the plan at Cambridge shows that it has great merits when those who administer it take the duty seriously, as Sir T. C. Allbutt has always done.

### A GENERAL POST OF CORONERS' DISTRICTS.

An Order in Council has recently been issued making various changes in the London coroners' districts. In future, the boroughs of Paddington and St. Marylebone are transferred from the central to the western coroners' districts; the parish of St. Luke, Finsbury, is transferred from the north-eastern to the central coroner's district; and where necessary the boundaries of the various coroners' districts are adjusted so that they will coincide with the boundaries of the Metropolitan boroughs. So confused, generally speaking, are our various local authorities and areas for special purposes that any attempt at simplification can always be termed by an interest opposing it as likely only to add to the present confusion. This is the penalty we pay for our often boasted habit of "muddling through."

### THE PRIMATE AND HOSPITAL SUNDAY.

JUDGING by his speech at the Mansion House a few nights ago, it would seem as if the Archbishop of Canterbury had been studying with some effect the Lord Mayor's article on Hospital Sunday which appeared in THE HOSPITAL SUNDAY SPECIAL NUMBER this year. In an admirable public appeal the Lord Mayor remarked that Hospital Sunday had been the inspiration of many ancillary movements, and referred especially to the League of Mercy in this respect. The Primate, however, complained at the Mansion House of the excessive superabundance of superfluous societies which had arisen, he said, on every side of church activity. So much had this impressed his Grace that he went on to say: "A new terror has now arisen, for each movement wants a Sunday of its own—Temperance Sunday, Peace Sunday, Empire Sunday, Sanitation Sunday, Citizen Sunday, Labour Sunday, Anti-vivisection Sunday," to say nothing of "Flower Sundays, and even in some churches, Pudding Sundays and Boot Sundays." We ourselves noted, by the way, a marmalade service only recently. But the point is how, ever and anon, the Primate seemed on the verge of mentioning Hospital Sunday, only to draw back again and again. "Sanitation Sunday" and "Anti-vivisection Sunday" reveal the temptation just as "Labour Sunday" and the rest betray his stern resistance to it. What was the psychology of this hesitation of the Archbishop's? It was, no doubt, the feeling common to us all that "Hospital Sunday" is not as these somewhat sad and crazy Sabbaths. Why? To say that its aim is a higher one would be uncharitable, and merely to beg the question. The true answer is that, as the Lord Mayor pointed out in THE HOSPITAL, Hospital Sunday is the parent, or, we would prefer to say, the predecessor of all these. A clergyman's suggestion, with a definite Christian sanction, Hospital Sunday was the fine idea inspired by a fine cause. Those mentioned by the Archbishop are its imitators. It is striking thus to find a medical Lord Mayor claiming, quite justly, Hospital Sunday as an inspiration of many good things, and the Archbishop of Canterbury bearing witness to Hospital Sunday's influence by recounting the less noble but inevitable side issues of its productivity.

### POINTS FOR THE PUBLIC ON SECRET REMEDIES.

WEIGHTY evidence against the traffic in quack medicines was given at the last sitting of the Select Committee by Mr. G. F. Harrison, B.Sc., F.I.C., the analyst who was employed by the British Medical Association to make the analyses of the proprietary medicines reported upon in "Secret Remedies." Mr. Harrison gave instances of medicines for which false claims were made, and of some which contained worthless, and others which contained dangerous ingredients. As an instance of a misleading statement he mentioned a cough pill which is advertised as being "free from opium" but which contains morphine, the active principle of opium; another instance is that of an advertised headache powder which is said to contain several ingredients but which, on analysis, is found to consist of 96 per cent. of acetanilide. He next mentioned examples of proprietary medicines the composition of which had been altered, stating that Powell's Balsam and Winslow's Soothing Syrup no longer contain morphine. He said that Daisy Powders were put up in two forms similarly labelled but differing in composition. Mr. Harrison admitted that certain vegetable compounds were incapable of analysis. Among articles the selling price of which is excessive in comparison with the cost he mentioned one which sells at 42s. though the estimated cost of the ingredients is 1s 0¾d., another which sells at 13s. of which the estimated cost of the ingredients is 3d., and another which sells at 50s. of which the estimated cost of the ingredients as 2½d. These figures should convince the lay public that the advertisements which produce the headache that demands the pill are themselves an expensive item in its cost.

### THE ROYAL SOCIETY OF MEDICINE.

A DISTINGUISHED panel of officers has been elected for the session 1912-13, to enter into office at the Royal Society of Medicine on October 1 next. The new president is Sir Francis H. Champneys, Bart., M.D., the well-known obstetrician of St. Bartholomew's. The honorary treasurers are Sir William Selby Church, Bart., M.D., and Sir Henry Morris, Bart., F.R.C.S., representing respectively medicine and surgery. The honorary librarians are Sir Rickman J. Godlee, Bart., M.S., and Norman Moore, M.D.; and the honorary secretaries are Mr. Herbert S. Pendlebury, F.R.C.S., and E. Farquhar Buzzard, M.D.

### THIS WEEK'S DRUG MARKET.

BUSINESS continues rather quiet, but there are signs that an improvement may set in before long. Several price changes have taken place, notably a reduction in the price of codeine. Cape aloes tends dearer, as also does balsam tolu. Buchu leaves are likely to be advanced in price. Stocks of manna are small and consequently the price is higher. Camphor is tending upwards in value. Gentian-root is scarce and dear. There is a probability of a further advance in the price of rhubarb, and the present would appear to be a good time to buy. Quicksilver has again advanced in price. Slight advances in the prices of borax and boric acid have been announced.

### TO OUR READERS.

CONTRIBUTIONS are specially invited from any of our readers to these columns. They should deal with topical subjects and news. They must be authenticated for the information of the Editor only. The minimum payment if published is 5s. There is no hard-and-fast rule as to space, but notes of about twenty lines in length are preferred.

